# The Protective Effects of Clams on Hypercholesterolemia in Late-Stage Triple-Transgenic Alzheimer’s Diseased Mice Hearts

**DOI:** 10.3390/md16080263

**Published:** 2018-08-01

**Authors:** You-Liang Hsieh, Hsu-Ju Teng, Yen-Hung Yeh, Cheng-Hong Hsieh, Chih-Yang Huang

**Affiliations:** 1 Department of Food Nutrition and Health Biotechnology, Asia University, 500, Lioufeng Road, Wufeng, Taichung 41354, Taiwan; hshsieh@asia.edu.tw (Y.-L.H.); dayeh806@yahoo.com.tw (C.-Y.H.); 2 Department of Medical Research, China Medical University Hospital, China Medical University, 2 Yude Road, Taichung 40447, Taiwan; 3 School of Health Diet and Industry Management, Chung Shan Medical University, 110, Jianguo N. Road, Sec. 1, Taichung 40201, Taiwan; hsuju@csmu.edu.tw; 4 Department of Nutrition, Chung Shan Medical University Hospital, 110, Jianguo N. Road, Sec. 1, Taichung 40201, Taiwan; 5 Graduate Institute of Basic Medical Science, China Medical University, 91, Hsueh-Shih Road, Taichung 40402, Taiwan; 6 Department of Chinese Medicine, China Medical University Hospital, 91, Hsueh-Shih Road, Taichung 40402, Taiwan

**Keywords:** clam, hypercholesterolemia, fatty acid, Alzheimer’s disease, cardiovascular disease

## Abstract

To investigate a high cholesterol diet in Alzheimer’s disease (AD) mice, they were fed with (2% cholesterol) in five groups with a control group, AD mice group, AD mice plus *Meretrix lusoria* group, AD mice plus *Geloina eros* group, and, AD mice plus *Corbicula fluminea* group for three months, and treated with the fatty acid profiles of clams by gas chromatography (GC). The results showed that treatment with clams for three months reduced Fas/L and Caspase-3 in the *Meretrix lusoria* and *Geloina eros* groups, but Fas-associated death domain (FADD) and Caspase-8 were strongly reduced in the *Geloina eros* group. For the mitochondria-dependent apoptotic pathway, the reduction of apoptosis proteins were observed in the hearts of clams-treated AD mice. BAK and Caspase-9 was reduced in the *Meretrix lusoria* group, but Caspase-3 and Cytochrome-*c* were reduced in *Geloina eros* group. Enhancement of survival proteins p-AKT, p-IGF1R, p-PI3K, Bcl-XL, Bcl2, and the longevity SIRT1 signaling proteins, p-AMPK-α, SIRT1, PGC1-α, p-FOXO3 were observed in clams-treated mice and even more strongly enhanced in the *Meretrix lusoria*, *Geloina eros* and *Corbicula fluminea* groups. This study observed that the ingestion of clams caused a reduction of apoptosis proteins and enhancement of survival and SIRT1 signaling proteins in the hearts.

## 1. Introduction

Cardiovascular disease shares some risk factors, and persons with Alzheimer’s disease (AD) have a higher inconsistency despite the overlapping risk of incident ischemic heart disease; however, the mechanisms involved in AD and cardiac cell apoptosis still remain unclear. Cholesterol is now considered for potential involvement in the pathogenesis of those human diseases that recognize hypercholesterolemia as primary risk factor or even a key causative factor including sporadic AD. Several epidemiological and experimental research studies have shown that high cholesterol levels (i.e., hypercholesterolemia) in plasma are an established risk driving force behind the development of AD and that lowering cholesterol levels through the use of statins can reduce this risk. However, it remains unclear how hypercholesterolemia is critical for neuronal function and may contribute to the onset and progression of AD pathology [1]. Recent studies have been confirmed the in vivo cumulation of fading cells with advancing age. Features of fading cells, such as the ability to revise their extracellular circumstance, could play a role in aging and age-related pathology [2]. Previous studies have indicated that AD [3], amyotrophic lateral sclerosis [4], sarcopenia [5], Parkinson’s disease [6], diabetes [7], cardiovascular disease [8], and cancer [9] are all age-related diseases.

AD is characterized by progressive cognitive decline usually beginning with impairment in the ability to form recent memories, as more than 30 million people suffer from this disease; furthermore, inevitably affecting all intellectual functions and leading to complete dependence for basic functions of daily life, and premature death [10]. Between 2000 and 2013, deaths from AD increased [11]. Therefore, we should pay attention to AD.

Cardiovascular disease (CVD) is a widespread disease has high incidence and death rate in the world [12]. Hypertrophy is a cardiovascular disease, which is divided into physiological and pathological hypertrophy. Subjected to short-term pressure will make the myocardial cells increase isometric contraction. When the pressure continues, it can make physiological hypertrophy become pathological hypertrophy. Pathological hypertrophy is divided into concentric hypertrophy and eccentric hypertrophy. Concentric hypertrophy could make myocardial thicken and increase contractility of the heart, while eccentric hypertrophy could make myocardial elongate. In late-stage pathological hypertrophy that caused myocardial apoptosis, this became heart failure [13]. One school of thought is that cholesterol plays a role and therefore statins are used. The second school of thought is that inflammation, and not cholesterol, is a cause of CVD [14]; clams have strong anti-inflammatory lipids as most marine sources [15] and it is the activities of these lipids that contribute to the anti-atherogenic properties of clams.

AD patients have a higher risk of accessary ischaemic heart disease [16]. Many studies demonstrated the involvement of cardiovascular disease-related pathways in AD [17]. AD and cardiovascular disease have the same risk factors [18]. Cardiovascular and carotid artery diseases are two major risk factors of AD [19]. Corder et al. [20] found the early-onset AD had little cardiovascular damage, and the late-onset AD had frequent heart valve damage, and evidence of ischemic damage to the left ventricular myocardium. In AD pathogenesis, brain hypoperfusion and microthrombi may conduce to the development of AD pathology or to the expression of dementia at an earlier stage.

A recent study showed that cardiac programmed cell apoptosis included two major signaling pathways, the apoptotic cell-death pathways. Apoptosis is induced via two main routes involving either the mitochondria (the intrinsic pathway) or the activation of death receptors [21]. In the death receptor-dependent apoptotic pathway, Fas/L could bind with Fas/R, and activate Fas-associated death domain (FADD), caspaes-8, and downstream Caspaes-3 induced apoptosis. When caspaes-8 makes Bid converted to tBid in the mitochondria and bind with Bad, Bak, and Bax, cytochrome is released from mitochondria to activate caspase-9. Furthermore, caspase-9 activated the downstream apoptosis path and finally induced cell death. In the survival pathway, p-IGF1-R could bind with the receptor, and then activate downstream p-PI3K, and p-AKT, that made Bad phosphorylation could leave the mitochondria, and then Bcl-2, and Bcl-xL expression could induce survival. SIRT1 has been implicated as an antiaging factor in numerous dysregulated physiologies that include glucose homeostasis, neurodegeneration, and mitochondrial integrity [22]. The signaling pathways of SIRT1 is a promising protective molecule to treat age-related CVDs, retard cardiovascular aging via inhibiting oxidative stress, delay cardiovascular aging by suppressing inflammation and slow cardiovascular aging through promotion of autophagy [23].

AD and cardiovascular disease share some risk factors and disease-related pathways, and persons with AD displayed a higher risk of ischemic heart disease. Hard clam, oyster and abalone are popular marine shellfish and traditionally used as a Chinese remedy for liver disease and chronic hepatitis in traditional folk medicine. Many researchers have revealed that seafood extracts have biological properties, such as antitumor and antihepatitis [24,25,26].

Omega-3 polyunsaturated fatty acids (n-3 PUFAs) mainly found in marine products lead to a low prevalence of cardiovascular disease (including myocardial infarction and sudden cardiac death, stroke and myocardial infarction) [27]. Animal studies have shown that n-3 PUFAs supplementation reduces amyloid-β and Tau pathology and improves cognition in animal models of AD [28]. There are two ways in which n-3 can be of significant benefit in the prevention and treatment of AD and other dementias by promoting neurite outgrowth [29,30]. The relationships between AD and dementia and fatty acids seem to be very complex. Many studies have found a correlation between lower PUFA levels and the risk of AD [31,32,33]. Therefore, in order to determine the role of hypercholesterolemia at various stages of triple-transgenic AD mice, in the present study the first part investigated the fatty acid profile of clams and evaluated the effects of a high cholesterol diet to inspect the heart functional changes, and the triple-transgenic AD mice were gastric-fed with *Geloina eros* extract (GEE), *Corbicula fluminea* extract (CFE) and *Meretrix lusoria* extract (MLE) for three months to study their effects in mechanisms to enhance-survival and anti-apoptotic effects.

## 2. Results

Fatty acid compositions of lipids extracted from the *Meretrix lusoria*, *Geloina eros* and *Corbicula fluminea* are shown in Table 1. For *Meretrix lusoria*, *Geloina eros* and *Corbicula fluminea*, polyunsaturated fatty acids (PUFA) in our study ranged from 50.63% to 60.08% of total fatty acid was dominant, followed by monounsaturated fatty acids (MUFA) from 20.66% to 28.96% of total fatty acid and saturated fatty acids (SFA) from 19.26% to 22.54% of total fatty acids. Palmitic acid (C16:0) from 7.65% to 12.31% of total fatty acid were the most prevalent saturated fatty acid, whilst docosahexaenoic acid (C22:6 n-3, DHA) from 9.21% to 12.21% of total fatty acid was dominant PUFA. The ratios of total n-3 PUFA to total n-6 PUFA were 1.13%, 1.40% and 1.30% of total fatty acid in the *Meretrix lusoria*, *Geloina eros* and *Corbicula fluminea*, respectively. Comparing PUFA among the *Meretrix lusoria*, *Geloina eros* and *Corbicula fluminea*, lipids from the *Geloina eros* showed the highest (*p* < 0.05) content 60.08% of total fatty acid, compared with the *Meretrix lusoria*, *Geloina eros* and *Corbicula fluminea*. However, the higher n-3 PUFA (*p* < 0.05) content was found in the *Geloina eros* and *Corbicula fluminea*, compared with that of the *Meretrix lusoria*. *Geloina eros* lipids constituted the highest (*p* < 0.05) content of n-6 PUFA, compared with the others. When considering eicosapentaenoic acid (C20:5 n-3, EPA) and docosahexaenoic acid (C22:6 n-3, DHA) in *Meretrix lusoria*, *Geloina eros* and *Corbicula fluminea*, it was found that lipids from the *Geloina eros* had the highest (*p* < 0.05) DHA content, whilst those from the *Geloina eros* contained the highest (*p* < 0.05) amounts of EPA. Additionally, the relatively high n-3/n-6 PUFA ratio indicated the high proportion of essential n-3 fatty acids in *Meretrix lusoria*, *Geloina eros* and *Corbicula fluminea*. Regarding MUFA, C18:1 n-9 was the dominant fatty acid in the *Meretrix lusoria*, *Geloina eros* and *Corbicula fluminea* from 5.13% to 8.59% of total fatty acid, followed by C20:1 n-11 from 2.13% to 3.65% of total fatty acid and C16:1 n-7 from 2.36% to 4.83% of total fatty acid. SFA content of the *Meretrix lusoria*, *Geloina eros* and *Corbicula fluminea* ranged from 19.26% to 22.54% of total fatty acid. However, *Meretrix lusoria*, *Geloina eros* and *Corbicula fluminea* showed a much higher PUFA content from 50.63% to 60.08% of total fatty acid.

To investigate whether *Meretrix lusoria*, *Geloina eros* and *Corbicula fluminea* could inhibit the death receptor-dependent apoptotic pathway in AD mice, the representative protein products of Fas receptor, FADD, Caspase-8, BAK, C-Caspase-9 and Caspase-3 extracted from the left ventricles of excised hearts in control mice, AD mice, AD + MLE mice, AD + GEE mice and AD + CFE mice were measured by Western blotting analysis. The results indicated that reduced expression of apoptosis proteins were observed in the hearts of AD + MLE, AD + GEE and AD + CFE mice. Moreover the reduction of Caspase-8 was more significant in the group of MLE treated mice (*p* < 0.05) compared with the AD group, but Fas receptor, FADD and Caspase-3 were strongly reduced in the group of AD + MLE, AD + GEE and AD + CFE mice (*p* < 0.05) compared with the AD group; moreover the reduction of BAK and C-Caspase-9 was more significant in the group of AD+MLE (*p* < 0.05) compared with the AD group, but the reduction of C-Caspase-3 and Cytochrome-*c* was more significant in the group of AD + GEE (Table 2).

To investigate whether *Meretrix lusoria*, *Geloina eros* and *Corbicula fluminea* could enhance the activation of the survival pathway in AD mice, the representative protein products of p-AKT, p-IGF1R, p-PI3K, Bcl-XL, and Bcl2 were extracted from the left ventricles of excised hearts in control, AD, AD + MLE, AD + GEE and AD + CFE mice were measured by Western blotting analysis. The results indicated that enhancement of survival proteins p-IGF1R, p-PI3K p-AKT, Bcl-XL, and Bcl2 were observed in the hearts of AD + MLE, AD + GEE and AD + CFE mice. Moreover, expression of these proteins was strongly enhanced in the group of AD + MLE and AD + GEE (*p* < 0.05) compared with the AD group (Table 3).

To investigate whether *Meretrix lusoria*, *Geloina eros* and *Corbicula fluminea* could activate SIRT1 pathway in AD mice, the representative protein products of p-AMPK-α, SIRT1, PGC1-α, and p-FOXO3 were extracted from the left ventricles of excised hearts in control, AD, AD + MLE, AD + GEE and AD + CFE mice were measured by Western blotting analysis. The results indicated that enhanced expression of SIRT1 signaling proteins, p-AMPK-α, SIRT1, PGC1-α and p-FOXO3 were observed in the hearts of AD + MLE, AD + GEE and AD + CFE mice. Moreover the enhanced expression of these proteins was more significant in the group of AD + GEE (*p* < 0.05) compared with the AD group (Table 4).

**Figure 1:**
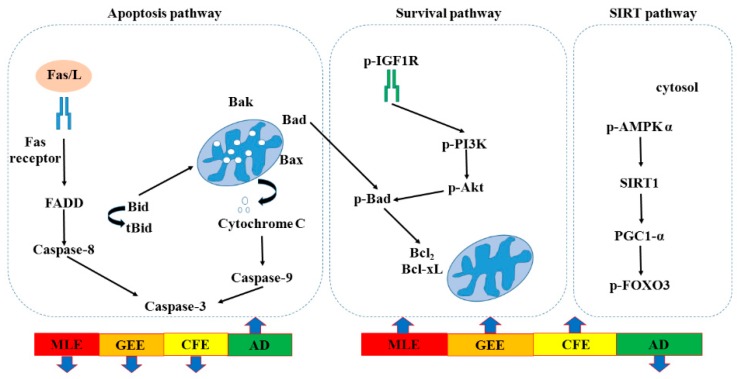
Apoptotic and survival pathway.

In death-receptor dependent apoptotic pathway, FAS/L, FADD and C-Caspase-3 were strongly reduced in MLE and GEE treatment, but Caspase-8 was strongly reduced in GEE treatment. For the mitochondria-dependent apoptotic pathway, the reduction of apoptosis proteins were observed in the hearts of MLE, GEE and CFE treatment. BAK and C-Caspase-9 were reduced in the MLE treatment, but Cytochrome-*c* was reduced in GEE treatment. Enhancement of survival proteins p-IGF1R, p-PI3K p-AKT, Bcl-XL, Bcl2 and longevity SIRT1 signaling proteins, p-AMPK-α, SIRT1, PGC1-α, p-FOXO3 were observed in the MLE, GEE and CFE treatment and even more strongly enhanced in the MLE and GEE treatment (*p* < 0.05) compared with the AD group (Figure 1).

## 3. Discussion

Apoptosis is a process of programmed cell death in which cells activate an intrinsic suicide program to self-destruct. This process plays a major role in development and which ensures tissue homeostasis, and safeguards the organism by eliminating unnecessary and unwanted cells, or cells that may constitute some form of apoptosis are associated with a variety regulation of human diseases [34]. It is adjusted by the interaction between survival and apoptotic pathways, with change the balance between the two pathways determining cell fate [35]. The apoptotic pathway exhibits increased levels with a reduced survival pathway in heart disease [36]. Jiang et al. [37] demonstrated that the numbers of apoptotic cells increased in the rats that were fed a high-fat diet for 12 and 16 weeks and fatty acids cause changess in death and survival pathways and eventually apoptosis [38,39]; EPA and DHA reduce the risk of breast cancer and modulates breast carcinogenesis through regulation of the apoptotic pathway prevention of breast cancer development [40]. In this study, the expression of C-Caspase-3 and C-Caspase-9 in hearts increased in AD mice, which is also an important factor in the cell apoptosis. This can be reduced by treatment with clams. *Meretrix lusoria*, *Geloina eros* and *Corbicula fluminea* contain a high amount of essential fatty acids, especially n-3 PUFAs, which can inhibit proinflammatory cytokine activity [41], which could be beneficial to health. Because a diet low in seafood n-3 PUFAs is reported as a contributor to ischemic heart disease disability-adjusted life-years and is considered a dietary risk factor with potentially significant effects on mortality worldwide [42,43], authoritative bodies recommend intake of EPA and DHA for heart and overall health [44,45].

The cardiac survival pathway is mediated by the IGF-1, IGF receptor, p-PI3K, p-Akt, Bcl-2 and Bcl-xL, which in turn blocks cardiacmyocyte apoptosis [46]. In the heart, the expression of the survival pathway upstream molecule p-IGF1R was reduced in AD mice. After adding MLE, GEE and CFE, p-IGF1R expression increased. The expression of downstream molecules p-PI3k and p-Akt increased when AD mice were treated with MLE, GEE and CFE (Table 3). SIRT1 increased cellular stress resistance by increasing insulin sensitivity. A decrease in circulating free fatty acid sand insulin-like growth factors (IGF1) increased AMPK activity and PGC-1α activity in mitochondria [47]. Our experimental data also showed similar results. In the AD + MLE, AD + GEE and AD + CFE groups, p-IGF1R and SIRT1expression increased.

In conclusion, the IGF1R/PI3K/AKT survival pathway in the AD mice heart can be increased through treatment with MLE, GEE and CFE. As the AD mice lost the heart function, this study showed that SIRT1 and its downstream PGC-1α constitute a novel alternative survival pathway for the heart. With increasing age and compensatory performance, exercise training can increase SIRT longevity pathway performance instead of IGF1 survival signaling and increase the chance for cardiomyocytes survival. Clams could inhibit heart damage via promoting cardiac cell survival and preventing apoptosis in AD mice.

## 4. Materials and methods

### 4.1. Materials

All other chemicals used were reagent grade in the purest form available commercial source.

### 4.2. Preparation of Clams

*Geloina eros, Corbicula fluminea* and *Meretrix lusoria* were collected from the Zeng Jiechong aquaculture farm (Yulin, Taiwan), *Corbicula fluminea* was provided by the aquaculture farm (Hualien, Taiwan), Taiwan. The crude extract was prepared as follows: tissue from fresh *Geloina eros, Corbicula fluminea* and *Meretrix lusoria* (5000 g) was cut into small pieces, homogenized in a blender and extracted with 5.0 L ethyl acetate. This procedure was repeated three times. The resulting supernatant extract was filtered and concentrated by rotary evaporator working under a vacuum and then freeze-dried; the yield of *Geloina eros* extract (GEE), *Corbicula fluminea* extract (CFE) and *Meretrix lusoria* extract (MLE) powder was 10.0 g for the experiments and was stored at −18 °C. (clams, on 8–10% proteins, 1–3% fats etc.)

### 4.3. Animal Test

This study was performed in five groups, each of which consisted of eight mice. Sixteen-month three gene knock out triple-transgenic Alzheimer’s disease (APPSWE-B6; SJL background) [Tg2576] mice were weighing between 50–65 g and randomly divided into five groups. Control group (fed a basal diet), AD group (fed a basal diet), AD plus GEE group, AD plus CFE group and AD plus MLE group, the components of all group diets are listed in Table 5 for three months. The body weight of the mice was assessed throughout the trial. The mice were given a carbon dioxide overdose at three months after the beginning of the trial. All animal protocol was approved by the Institutional Animal Care and Use Committee (IACUC) of Chung Shan Medical University (CSMU). Institutional and national guidelines for the care and use of animals were followed.

### 4.4. Determination of Fatty Acid Profiles

The fatty acid composition of GEE, CFE and MLE powder were extracted with diethyl ether, fatty acid methyl esters (FAMEs) were obtained through acid methylation with diazomethane and followed by acetylation with acetic anhydride produced from total lipid aliquots and methylated with boron trifluoride-methanol, determined using gas chromatography in a Thermo Trace GC 3300 model (Thermo Scientific, Waltham, MA, USA.) according to the method of Zelinkova et al. [48]. The temperatures of the injector and detector (FID) were set at 250 °C and 260 °C with nitrogen as the carrier gas respectively. The temperature program was programmed from 140 to 240 °C at a rate of 4 °C/min. High-purity helium was used as the carrier gas at a flow rate of 1 mL/min. The samples (1.0 μL) were manually injected into the injection port and identified FAs were presented as area percentage of total FAs. Individual fatty acids were expressed as percentage of total identified FAME, and were then categorized saturated fatty acid (SFA), monounsaturated (MUFA), and polyunsaturated fatty acids (PUFA).

### 4.5. Tissue Grinding

The collected left ventricular tissues of control, AD, AD + GEE, AD + CFE and AD + MLE groups were washed by Phosphate Buffered Saline (PBS) buffer and then weighted. About 0.1 g tissue was added by 1 mL lysis buffer. The mixture was homogenizing stirred 1200× *g* for 20 min under 4 °C. After stirring, we extracted clean suspension at the upper layer. The solution was stirred again with the same condition as the first stir to extract more clean suspension at the upper layer.

### 4.6. Lowry Protein Assay

Bovine serum albumin (BSA) as a standard and plotted the standard curve of different concentration of BSA (0 mg/mL, 0.1 mg/mL, 0.2 mg/mL, 0.3 mg/mL, 0.4 mg/mL, and 0.5 mg/mL). The samples were diluted to 40 times with a total volume 200 µL or 250 µL alkaline copper reagent (2% Na-K tartrate, 1% CuSO_4_·5 H_2_O, 2% Na_2_CO_3_ in 0.1 N NaOH (aq) and mixed in the *v*/*v*/*v* = 1/1/98) added to mix for 50 min at room temperature (RT). Folin–Ciocalteu phenol reagent was then added to mix for 10 min at RT. The absorbance at 750 nm was measured to determine the relative concentrations of the proteins [49].

### 4.7. Western Blotting

The protein sample (0.3 g) was homogenized in 3 mL of ice-cold PBS. Homogenates were centrifuged at 10,000× *g* for 10 min at 4 °C. The protein concentration of the supernatant was determined based on the Biuret reaction using a bicinchoninic acid (BCA) kit with bovine serum albumin as the standard. Ten microliters of protein was electrophoresed on a 12% sodium dodecyl sulfate (SDS)–polyacrylamide gel and transferred to membranes. Blots were probed with goat anti- sodium dodecyl sulfate (SOD) (1:1000) and rabbit anti-catalase (1:8000) and visualized with a 1:5000 dilution of IgG conjugated to horseradish peroxidase [50]. 

### 4.8. Statistics

The experimental data were shown by mean ± standard error of the mean (SEM) and the differences of the four groups were analyzed by Student’s *t* test. Multi-groups differences among the means were analyzed by one way analysis of variance (ANOVA). When *p* < 0.05, the difference was meaningful in statistics [51].

## Figures and Tables

**Table 1:** Fatty acid profiles of the *Meretrix lusoria* extract (MLE), *Geloina eros* extract (GEE) and *Corbicula fluminea* extract (CFE).

Fatty Acid	MLE (%)	GEE (%)	CFE (%)
Saturated fatty acid (SFA) (% of total fatty acid)			
C12:0	0.13 ± 0.02 ^a^	0.21 ± 0.03 ^b^	0.15 ± 0.02 ^a^
C14:0	0.75 ± 0.13 ^a^	1.23 ± 0.07 ^b^	0.95 ± 0.08 ^a^
C15:0	1.56 ± 0.22 ^c^	0.53 ± 0.10 ^a^	0.87 ± 0.12 ^b^
C16:0	12.31 ± 1.56 ^b^	7.65 ± 1.23 ^a^	10.56 ± 1.82 ^a^
C17:0	6.23 ± 0.35 ^b^	5.76 ± 0.52 ^b^	3.27 ± 0.32 ^a^
C18:0	0.21 ± 0.03 ^a^	0.31 ± 0.02 ^b^	0.39 ± 0.02 ^b^
C20:0	1.13 ± 0.06 ^a^	3.25 ± 0.07 ^c^	2.87 ± 0.05 ^b^
C23:0	0.22 ± 0.01 ^a^	0.32 ± 0.01 ^c^	1.35 ± 0.03 ^b^
Monosaturated fatty acid (MUFA) (% of total fatty acid)			
C14:1	0.51 ± 0.02 ^b^	0.36 ± 0.01 ^a^	1.56 ± 0.21 ^c^
C15:1	0.62 ± 0.03 ^b^	0.21 ± 0.02 ^a^	0.73 ± 0.01 ^c^
C16:1 n-7	3.52 ± 0.78 ^b^	2.36 ± 0.56 ^a^	4.83 ± 0.32 ^b^
C17:1	1.23 ± 0.02 ^c^	0.56 ± 0.03 ^b^	0.39 ± 0.01 ^a^
C18:1 n-11	2.32 ± 0.56 ^b^	1.21 ± 0.21 ^a^	3.26 ± 0.23 ^c^
C18:1 n-9	5.13 ± 1.21 ^a^	6.56 ± 1.65 ^a^	8.59 ± 1.58 ^b^
C18:1 n-7	1.16 ± 0.02 ^b^	2.22 ± 0.03 ^c^	0.25 ± 0.01 ^a^
C20:1 n-7	1.33 ± 0.01 ^a^	2.29 ± 0.01 ^c^	3.48 ± 0.02 ^b^
C20:1 n-9	1.25 ± 0.03 ^b^	0.65 ± 0.02 ^a^	2.36 ± 0.03 ^c^
C20:1 n-11	3.65 ± 0.16 ^b^	2.13 ± 0.11 ^a^	3.24 ± 0.35 ^b^
C22:1 n-9	0.26 ± 0.01 ^a^	2.11 ± 0.02 ^b^	0.27 ± 0.02 ^a^
Polyunsaturated fatty acid (PUFA) (% of total fatty acid)			
C16:3 n-3	1.72 ± 0.05 ^c^	1.21 ± 0.03 ^a^	1.33 ± 0.05 ^b^
C18:2 n-6	0.21 ± 0.01 ^a^	0.36 ± 0.01 ^b^	0.22 ± 0.02 ^a^
C18:3 n-3	3.23 ± 0.36 ^a^	5.63 ± 0.52 ^b^	3.23 ± 0.65 ^a^
C18:3 n-6	1.26 ± 0.13 ^b^	0.82 ± 0.18 ^a^	1.12 ± 0.15 ^b^
C18:4 n-3	1.03 ± 0.12 ^b^	0.75 ± 0.13 ^a^	1.09 ± 0.15 ^b^
C20:1 n-6	4.93 ± 0.23 ^a^	6.31 ± 0.56 ^b^	4.56 ± 0.62 ^a^
C20:3 n-6	1.15 ± 0.03 ^b^	0.53 ± 0.05 ^a^	2.02 ± 0.07 ^c^
C20:3 n-3	0.88 ± 0.02 ^c^	0.62 ± 0.03 ^b^	0.21 ± 0.02 ^a^
C20:4 n-6	6.52 ± 0.52 ^a^	7.53 ± 0.20 ^b^	5.33 ± 0.67 ^a^
C20:4 n-3	1.22 ± 0.02 ^b^	0.76 ± 0.03 ^a^	1.53 ± 0.06 ^c^
C20:5 n-3, EPA	7.32 ± 0.58 ^a^	8.50 ± 0.75 ^b^	6.58 ± 0.23 ^a^
C21:5 n-3	1.25 ± 0.06 ^b^	0.12 ± 0.02 ^a^	1.65 ± 0.01 ^c^
C22:4 n-6	3.23 ± 0.05 ^b^	4.52 ± 0.03 ^c^	2.25 ± 0.03 ^a^
C22:5 n-3	6.57 ± 0.08 ^c^	5.26 ± 0.07 ^b^	3.78 ± 0.07 ^a^
C22:5 n-6	5.73 ± 0.05 ^b^	4.95 ± 0.03 ^a^	6.52 ± 0.06 ^c^
C22:6 n-3, DHA	10.23 ± 0.05 ^b^	12.21 ± 0.08 ^c^	9.21 ± 0.03 ^a^
Sums of fatty acids			
ƩSFA	22.54 ± 1.56 ^b^	19.26 ± 1.52 ^a^	20.41 ± 1.23 ^a^
ƩMUFA	20.98 ± 2.31 ^a^	20.66 ± 3.68 ^a^	28.96 ± 2.23 ^b^
ƩPUFA	56.48 ± 3.62 ^b^	60.08 ± 2.35 ^c^	50.63 ± 2.57 ^a^
Ʃn-3	26.13 ± 1.23 ^b^	35.06 ± 1.61 ^b^	28.61 ± 1.20 ^a^
Ʃn-6	23.03 ± 0.51 ^b^	25.02 ± 0.31 ^c^	22.02 ± 0.22 ^a^
Fatty Acid	MLE (%)	GEE (%)	CFE (%)
Ratios			
PUFA/SFA	2.50 ± 0.56 ^a^	3.12 ± 0.52 ^b^	2.48 ± 0.35 ^a^
MUFA/SFA	0.93 ± 0.01 ^a^	1.07 ± 0.02 ^b^	1.42 ± 0.03 ^c^
DHA/EPA	1.40 ± 0.01 ^a^	1.44 ± 0.02 ^b^	1.40 ± 0.01 ^a^
n-3/n-6	1.13 ± 0.02 ^c^	1.40 ± 0.01 ^b^	1.30 ± 0.02 ^a^
n-6/n-3	0.88 ± 0.02 ^a^	0.71 ± 0.01 ^b^	0.77 ± 0.01 ^c^

(a–c) Values represent mean ± SD (*n* = 3). Different lowercase letters in the same row indicate significant difference (*p* < 0.05). ƩSFA = saturated fatty acid; ƩMUFA = mono-unsaturated fatty acid; ƩPUFA = poly-unsaturated fatty acid; Ʃn-3 = total omega 3 fatty acid; Ʃn-6 = total omega 6 fatty acid.

**Table 2:** Effect of MLE, GEE and CFE supplementation on heart death receptor-dependent apoptotic pathway (Fas receptor, FADD, Caspase-8, BAK, C-Caspase-9, Cytochrome-*c* and Caspase-3) mRNA expression in AD mice.

Enzyme	Enzyme/β-actin				
	Control	AD	AD + MLE	AD + GEE	AD + CFE
Fas receptor	1.00 ± 0.15 ^b^	2.65 ± 0.15 ^a^	1.62 ± 0.18 ^c^	1.65 ± 0.28 ^c^	1.86 ± 0.13 ^c^
FADD	1.00 ± 0.13 ^b^	2.42 ± 0.13 ^a^	1.53 ± 0.15 ^c^	1.45 ± 0.12 ^c^	1.77 ± 0.28 ^c^
Caspase-8	1.00 ± 0.08 ^b^	2.75 ± 0.08 ^a^	1.78 ± 0.27 ^c^	1.56 ± 0.18 ^c^	1.89 ± 0.16 ^c^
BAK	1.00 ± 0.07 ^b^	2.21 ± 0.07 ^a^	1.35 ± 0.16 ^c^	1.33 ± 0.19 ^c^	1.45 ± 0.08 ^c^
C-Caspase-9	1.00 ± 0.09 ^b^	2.33 ± 0.09 ^a^	1.56 ± 0.38 ^c^	1.68 ± 0.17 ^c^	1.82 ± 0.28 ^c^
Cytochrome-*c*	1.00 ± 0.19 ^b^	2.41 ± 0.89 ^a^	1.56 ± 0.37 ^c^	1.42 ± 0.35 ^c^	1.75 ± 0.33 ^c^
Caspase-3	1.00 ± 0.32 ^b^	2.56 ± 0.89 ^a^	1.46 ± 0.21 ^c^	1.62 ± 0.28 ^c^	1.88 ± 0.19 ^c^

All the data showed here are the ratios of each enzyme to β-actin according to the quantitative and statistical results of reverse transcription-polymerase chain reaction (RT-PCR) from densitometric analysis. (a–c) Values represent mean ± SD (*n* = 8), and values different superscripts are significantly different (*p* < 0.05). AD, Alzheimer’s disease; MLE, *Meretrix lusoria*; GEE, *Geloina eros*; CFE, *Corbicula fluminea*.

**Table 3:** Effect of MLE, GEE and CFE supplementation on heart survival pathway (p-IGF1R, p-PI3K p-AKT, Bcl-XL and Bcl2) mRNA expression in AD mice.

Enzyme	Enzyme/β-actin				
	Control	AD	AD + MLE	AD + GEE	AD + CFE
p-IGF1R	1.00 ± 0.21 ^b^	0.76 ± 0.13 ^c^	3.63 ± 0.36 ^a^	3.91 ± 0.55 ^a^	2.32 ± 0.88 ^a^
p-PI3K	1.00 ± 0.15 ^b^	0.58 ± 0.15 ^c^	3.37 ± 0.37 ^a^	2.75 ± 0.65 ^a^	1.63 ± 0.52 ^a^
p-AKT	1.00 ± 0.16 ^b^	0.35 ± 0.15 ^c^	2.55 ± 0.32 ^a^	2.83 ± 0.53 ^a^	1.82 ± 0.27 ^a^
Bcl-XL	1.00 ± 0.22 ^b^	0.25 ± 0.29 ^c^	1.32 ± 0.27 ^a^	1.36 ± 0.17 ^a^	1.25 ± 0.15 ^a^
Bcl2	1.00 ± 0.11 ^b^	0.56 ± 0.31 ^c^	1.35 ± 0.12 ^a^	1.38 ± 0.13 ^a^	1.26 ± 0.13 ^a^

All the data showed here are the ratios of each enzyme to β-actin according to the quantitative and statistical results of RT-PCR from densitometric analysis. (a–c) Values represent mean ± SD (*n* = 8), and values different superscripts are significantly different (*p* < 0.05). AD, Alzheimer’s disease; MLE, *Meretrix lusoria*; GEE, *Geloina eros*; CFE, *Corbicula fluminea*.

**Table 4:** Effect of MLE, GEE and CFE supplementation on heart SIRT1 pathway (p-AMPK-α, SIRT1, PGC1-α and p-FOXO3) mRNA expression in AD mice.

Enzyme	Enzyme/β-actin				
	Control	AD	AD + MLE	AD + GEE	AD + CFE
p-AMPK-α	1.00 ± 0.13 ^b^	2.56 ± 0.12 ^a^	1.23 ± 0.39 ^b^	1.33 ± 0.27 ^b^	1.23 ± 0.65 ^b^
SIRT1	1.00 ± 0.22 ^b^	2.21 ± 0.23 ^a^	3.56 ± 0.65 ^b^	4.46 ± 0.52 ^b^	3.21 ± 0.53 ^b^
PGC1-α	1.00 ± 0.35 ^b^	2.65 ± 0.35 ^a^	1.33 ± 0.29 ^b^	1.43 ± 0.27 ^b^	1.31 ± 0.15 ^b^
p-FOXO3	1.00 ± 0.16 ^b^	2.36 ± 0.27 ^a^	1.23 ± 0.19 ^b^	1.35 ± 0.25 ^b^	1.13 ± 0.33 ^b^

All the data showed here are the ratios of each enzyme to β-actin according to the quantitative and statistical results of RT-PCR from densitometric analysis. (a–c) Values represent mean ± SD (*n* = 8), and values different superscripts are significantly different (*p* < 0.05). AD, Alzheimer’s disease; MLE, *Meretrix lusoria*; GEE, *Geloina eros*; CFE, *Corbicula fluminea*.

**Table 5:** Composition of the experimental diets for animal diet.

Ingredient	Diets				
	Control	AD (%)	AD + MLE (%)	AD + GEE (%)	AD + CFE (%)
Casein	20	20	20	20	20
Methionine	0.3	0.3	0.3	0.3	0.3
Cellulose	5	5	5	5	5
Corn oil	2	2	2	2	2
Cholesterol	2	2	2	2	2
MLE	0	0	6	0	0
GEE	0	0	0	6	0
CFE	0	0	0	0	6
Choline	0.2	0.2	0.2	0.2	0.2
Mineral mix^a^	3.5	3.5	3.5	3.5	3.5
Vitamin mix^b^	1	1	1	1	1
Corn starch	31	31	25	25	25
Sucrose	35	35	35	35	35

(a) Mineralsper 100 g diet: NaCl 7.4 g, K_2_C_6_H_5_O_7_·H_2_O 22g, K_2_SO_4_ 5.2 g, CaHPO_4_ 50 g, MgO 2.4 g, FeC_6_H_5_O_7_·5H_2_O 0.6 g, MnCO_3_ 0.35 g, CuCO_3_ 30 mg, CrK(SO_4_)_2_·12H_2_O 55 mg, CoCl_2_·6H_2_O 10 mg, KI 1 mg, ZnCO_3_ 160 mg. (b) Vitamin per 100 g diet: thiamine 100 mg, riboflavin 150 mg, pyridoxine HCl 100mg, nicotinamide 1000 mg, d-panthenate 500 mg, folic acid 50 mg, vitamine B_12_ 0.1 mg, vitamin A 2.5 × 10^5^ IU, vitamin E 100 mg, calciferol 2 × 10^4^ IU, vitamin C 3.7 × 10^3^ mg.

## Abbreviations

The following abbreviations are used in this manuscript:

AD	Alzheimer’s disease
AKT	Protein kinase B
AMPK	5’ AMP-activated protein kinase
ANOVA	Analysis of variance
Bak	Bcl2-antagonist/killer
Bcl2	B-cell lymphoma 2
Bcl-XL	B-cell lymphoma-extra large
BSA	bovine serum albumin
Caspase	cysteine-aspartic proteases
CFE	*Corbicula fluminea* extract
CVD	Cardiovascular disease
DHA	Docosahexaenoic acid
EPA	Eicosapentaenoic acid
FADD	Fas-associated death domain
FADD	Fas-associated death domain
FAMEs	Fatty acid methyl esters
Fas/L	Fas ligand
GC	gas chromatography
GEE	*Geloina eros* extract
IGF1	Insulin-like growth factor 1
IGF1R	Insulin-like growth factor 1 receptor
MLE	*Meretrix lusoria* extract
MUFA	Monounsaturated fatty acid
PI3K	Phosphoinositide 3-kinase
PUFA	polyunsaturated fatty acids
SFA	Saturated fatty acid
